# Mitral valve calcific embolization leading to myocardial infarction in a patient with end-stage renal disease

**DOI:** 10.1093/omcr/omag094

**Published:** 2026-06-08

**Authors:** Yousef Hailan, Nazar Mohammad, Hakam Alzaeem, Hiba Habib, Mhd Baraa Habib

**Affiliations:** Cardiology Department, Heart Hospital, Hamad Medical Corporation, Al Rayyan Road, Hamad Medical City, Doha, P.O. Box 3050, Qatar; Cardiology Department, Heart Hospital, Hamad Medical Corporation, Al Rayyan Road, Hamad Medical City, Doha, P.O. Box 3050, Qatar; Cardiology Department, Heart Hospital, Hamad Medical Corporation, Al Rayyan Road, Hamad Medical City, Doha, P.O. Box 3050, Qatar; Internal Medicine Department, Damascus University Hospital, Damascus, Syria; Cardiology Department, Heart Hospital, Hamad Medical Corporation, Al Rayyan Road, Hamad Medical City, Doha, P.O. Box 3050, Qatar

**Keywords:** Coronary embolism, End-stage renal disease, Tertiary hyperparathyroidism, Mitral valve calcification, Acute coronary syndrome, AngioVac

## Abstract

While atherothrombosis accounts for the majority of acute coronary syndrome (ACS) cases, non-atherosclerotic mechanisms like coronary embolism are important considerations in end-stage renal disease (ESRD). A 52-year-old woman with ESRD and tertiary hyperparathyroidism presented with myocardial infarction. Echocardiography identified mobile mitral valve masses. Angiography showed a left anterior descending artery occlusion, but intravascular ultrasound revealed minimal plaque burden. Histopathological analysis of aspirated material confirmed calcified necrotic debris rather than thrombus. Despite concurrent COVID-19 infection, the rigid mechanical behavior of the masses during attempted percutaneous aspiration distinguished the pathology from infective endocarditis. This case highlights calcific coronary embolism as a rare ACS cause in ESRD patients. Multimodal imaging and histopathology are crucial for accurate diagnosis.

## Introduction

While atherothrombosis causes majority of Acute Coronary Syndrome (ACS) cases, non-atherosclerotic mechanisms are increasingly recognized yet overlooked. These alternative mechanisms-including spontaneous coronary artery dissection, vasculitis, and coronary embolism- are important diagnostic considerations, particularly in younger patients or those lacking traditional risk factors [1–4].

Coronary embolism, although uncommon, can arise from various sources, including cardiac masses, valvular vegetations, and atrial thrombi [5, 6]. End-Stage Renal Disease (ESRD) is a significant risk factor for such embolic events. The metabolic derangement in CKD accelerates vascular and valvular calcification, creating a substrate for embolization [7, 8]. Mitral and aortic valve calcification has been observed in 45% and 34% of ESRD patients, respectively, rates 9- to15-fold higher than in the general population [8].

Differentiating these etiologies is important, as treatment approaches for embolic ACS differ from standard atherothrombotic management. We present a case of calcific embolization from degenerative mitral valve disease mimicking typical ACS, highlighting the necessity of considering non-atherosclerotic causes in high-risk populations.

## Case presentation

A 52-year-old woman presented with one day of central, non-radiating chest pain. Review of systems was notable for a new cough for 3 days. Her medical history included hypertension, IgA nephropathy leading to ESRD (peritoneal dialysis), and tertiary hyperparathyroidism (previously declining parathyroidectomy).

Examination revealed elevated jugular venous pressure and bilateral basal crackles. The ECG showed no acute ischemic changes, but high- sensitivity troponin was significantly elevated, rising from 396 ng/l on presentation to a peak of 14 279 ng/l. Mineral bone disease parameters consistent with severe tertiary hyperparathyroidism included PTH 2121 pg/ml (normal 15–65), serum calcium 2.15 mmol/l (normal 2.2–2.6), and serum phosphorus 4.17 mmol/l (normal 0.8–1.5).

**Figure 1:**
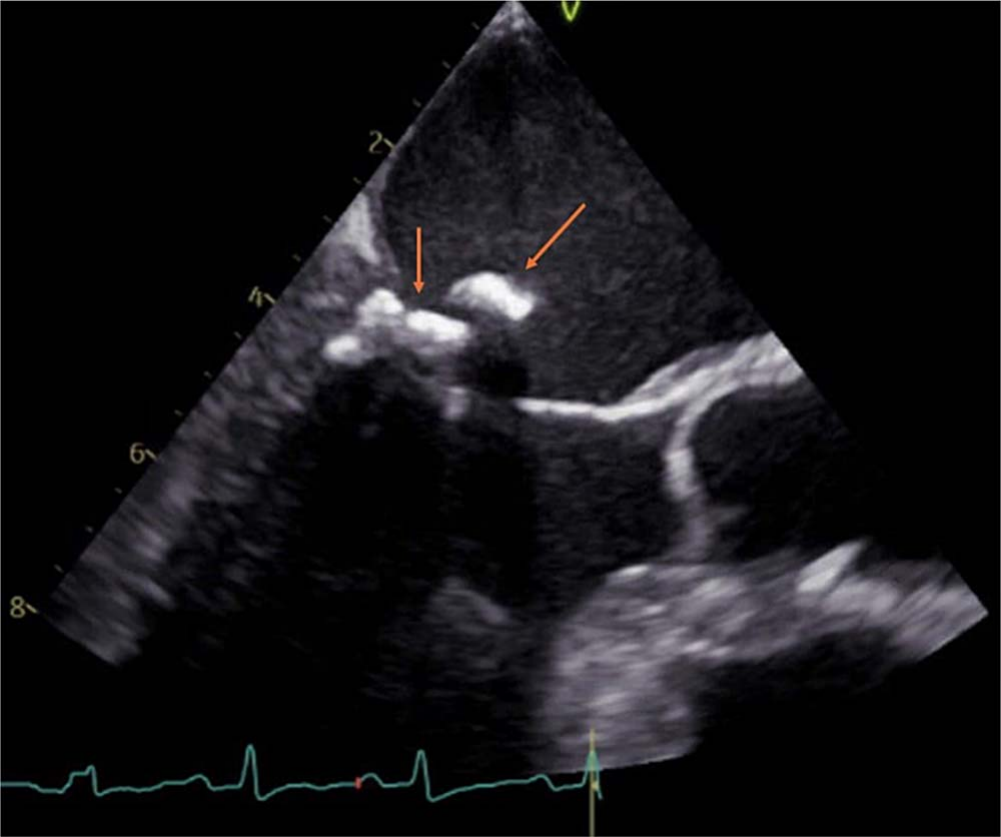
Transesophageal echocardiography (TEE). Mid-esophageal long-axis view demonstrating degenerative mitral valve disease with two mobile, calcified masses (arrows) attached to the posterior leaflet. The larger mass (1.4 × 1.0 cm) is attached to the P2 scallop, while a smaller mass (1.0 × 0.5 cm) is attached to the P1 scallop.

The patient was admitted as a high-risk Non-ST-Elevation Myocardial Infarction. Transthoracic echocardiography (TTE) showed reduced left ventricular systolic function (LVEF 41%), and revealed a 10 × 6 mm calcified, mobile mass on the atrial surface of the posterior mitral valve leaflet (Video Clip 1). Transesophageal echocardiogram (TEE) confirmed degenerative mitral valve disease with two mobile calcific masses on the posterior leaflet: a larger mass (1.4 × 1 cm) attached to P2 scallop, and a smaller mass (1 × 0.5 cm) attached to P1 scallop (Fig. 1), associated with mild mitral regurgitation. No significant aortic valve calcification, complex aortic atheroma, intracardiac thrombus, or left atrial appendage thrombus was identified on transesophageal echocardiography, making alternative embolic sources unlikely.

Coronary angiography revealed a 90% occlusion in the proximal left anterior descending (LAD) artery with an appearance suggestive of thrombus. Aspiration was performed, resulting in partial removal and distal embolization of the remaining material. The distal vessel was successfully treated with balloon angioplasty (TIMI-3 flow). Intravascular ultrasound (IVUS) was performed following aspiration thrombectomy and restoration of distal flow. IVUS demonstrated minimal underlying atherosclerotic plaque burden in the proximal LAD and no features of plaque rupture, plaque erosion, or calcified nodule, supporting an embolic mechanism rather than primary atherothrombotic disease. (Video Clips 2, 3).

Histopathological analysis of the aspirated fragments (0.2 × 0.2 × 0.1 cm aggregate) revealed calcified and necrotic material, rather than fibrin-rich thrombus.

Differentiating infective endocarditis from calcific degeneration was challenging. The patient was afebrile with negative blood cultures. However, inflammatory markers were elevated (CRP 152 mg/l [normal 0–5], Procalcitonin 2.90 ng/ml), and PET-CT showed FDG uptake at the mitral valve suspicious for infection. This inflammatory picture was eventually attributed to concurrent COVID-19 infection (PCR CT value 26.02) and right middle lobe pneumonia.

Given the uncertainty, the Heart Team opted for a percutaneous AngioVac aspiration. Under general anesthesia, TEE and fluoroscopic guidance, a 26-Fr cannula was positioned near the mitral masses through trans-septal access. Despite multiple attempts, flow rates could not exceed 2 L/min because the masses repeatedly obstructed the funnel.

The masses remained intact and resisted fragmentation by a 15 mm snare. This mechanical behavior provided compelling diagnostic evidence: the rigidity and obstruction were characteristic of heavy calcification rather than the friable texture of infective vegetations.

The patient completed a 4-week course of broad-spectrum antibiotics for presumptive pneumonia/endocarditis and was discharged. At 6-month follow-up, the previously mobile mitral mass had become fixed to the leaflet. At 18 months, the patient remained asymptomatic from a cardiac standpoint. Despite this embolic event, she continues to decline parathyroidectomy.

## Discussion

This case highlights the importance of distinguishing calcific coronary embolism from varying etiologies of ACS. While classic atherothrombosis is the default diagnosis for STEMI/NSTEMI, failing to identify a calcific embolic source can lead to inappropriate management. In this patient, the absence of proximal plaque on IVUS and the retrieval of ‘necrotic calcified material’ were the findings that shifted the diagnosis from plaque rupture to embolic phenomenon.

Although the mitral masses appeared mechanically rigid during AngioVac manipulation and resisted fragmentation, this does not exclude embolization. Calcific valvular lesions may undergo surface micro-fragmentation, whereby small calcified particles detach from the lesion surface due to repetitive leaflet motion and hemodynamic shear stress. The small size of the aspirated fragment in our case (0.2 cm aggregate) supports this mechanism, suggesting micro-embolization rather than detachment of the entire mass. Similar mechanisms have been described in embolization from degenerative calcific valvular lesions and cardiac calcifications [9].

The etiology of this event is due to the metabolic derangements associated with End-Stage Renal Disease (ESRD). Patients on renal replacement therapy face significantly elevated cardiovascular mortality [10–13]. The disruption of the calcium-phosphate-parathyroid hormone (PTH) axis—specifically hyperphosphatemia and secondary/tertiary hyperparathyroidism—drives extra-skeletal osteoblastic transformation [9]. This results in accelerated valvular calcification, creating a substrate for embolization [14]. In our patient, the long-standing refusal of parathyroidectomy likely allowed for unchecked tertiary hyperparathyroidism, directly contributing to the formation of the mobile, calcified mitral masses.

A challenge in this case was distinguishing these calcific masses from infective endocarditis. The possibility of infective endocarditis was carefully evaluated. Blood cultures remained persistently negative, there were no peripheral stigmata of endocarditis, and the echocardiographic findings were considered more consistent with degenerative calcific lesions than with vegetations. Although FDG uptake on PET-CT and elevated inflammatory markers raised concern for infection, these findings were likely attributable to concurrent COVID-19 infection and pneumonia. Overall, the clinical, microbiological, imaging, and histopathological findings favored a non-infective degenerative calcific process over infective endocarditis.

Here, the procedural behavior of the masses offered diagnostic insight, complementing the histopathology. During the AngioVac extraction attempt, the masses exhibited rigid mechanical resistance and repeatedly obstructed the funnel without fragmenting. This is highly distinct from the friable texture typical of infectious vegetations. Although 10–20% of endocarditis cases are culture-negative [15], the constellation of rigid mechanical properties, negative microbiology, and calcific histology supported a degenerative etiology.

The diagnostic picture was further clouded by concurrent COVID-19 infection. The viral infection likely drove the elevation in inflammatory markers (CRP, procalcitonin) and PET-CT metabolic activity, mimicking a bacterial infectious process. Additionally, the known prothrombotic and endothelial effects of COVID-19 [16], may have acted as trigger, facilitating the detachment from the pre-existing calcific mass.

To our knowledge, this is among the first documented cases of coronary embolization specifically from mitral valve calcific masses leading to myocardial infarction in an ESRD patient. While aortic sources are more commonly described, this case underscores that the mitral valve can be a source of calcific emboli.

Two key lessons learned: First, histopathological analysis of aspirated coronary material is invaluable in atypical ACS, as it provided the diagnosis here. Coronary embolism should therefore be considered in patients with ACS and minimal coronary atherosclerosis, particularly when structural cardiac lesions or severe metabolic disorders such as ESRD are present. Second, aggressive management of tertiary hyperparathyroidism is important to prevent cardiovascular calcifications in this population. This case is an example of the value of the Heart Team approach—utilizing multimodality imaging, new interventional techniques (AngioVac), and pathology—to guide management of complex presentations in high-risk patients.

Nevertheless, given the diagnostic uncertainty and potentially catastrophic consequences of untreated endocarditis in an immunocompromised ESRD patient, a complete 4-week antibiotic course was administered as recommended by the infectious disease team. This cautious approach is supported by literature when endocarditis cannot be definitively excluded in high-risk patients [17, 18].

## Supplementary Material

Video_1_2CH_omag094

Video_2_Pre-Intervention_omag094

Video_3_Post-Intervention_omag094

## Data Availability

The data supporting the findings of this case report are available within the manuscript and its supplementary materials. Additional data are available from the corresponding author upon reasonable request.
